# Supplementary figures and images for: Comparative Genome Analysis of ‘Candidatus Phytoplasma luffae’ Reveals the Influential Roles of Potential Mobile Units in Phytoplasma Evolution
Source: Front Microbiol. 2022 Feb 28;13:773608. doi: 10.3389/fmicb.2022.773608 (PMC8923039; doi:10.3389/fmicb.2022.773608)

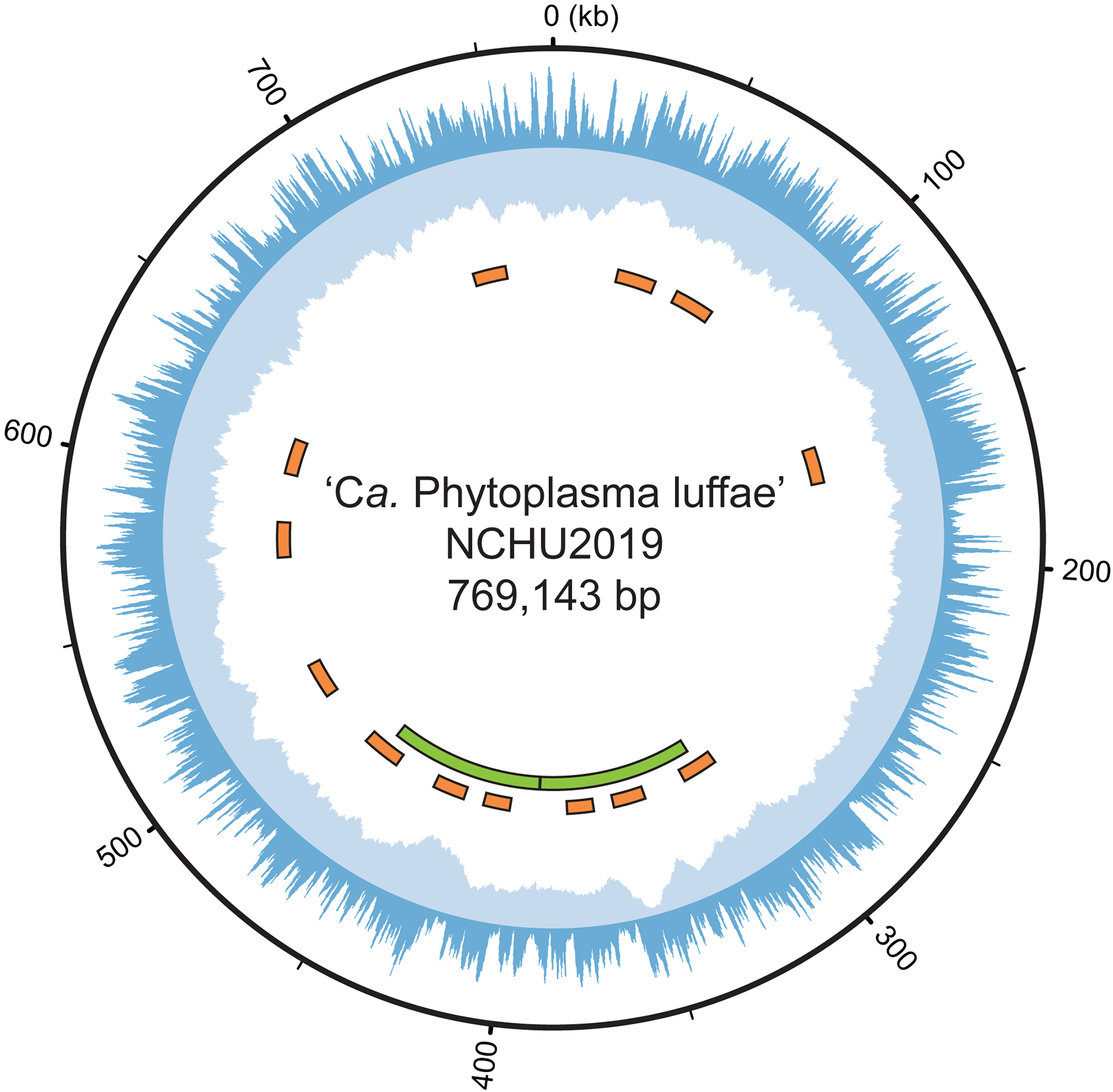

Supplement: Supplementary Figure 1 — Coverage pattern of raw read mapping results to the genome assembly. Rings from outside in: (1) Scale marks (kb). (2) Sequencing depth calculated from Illumina (dark blue; outward) and ONT (light blue; inward) raw reads mapped to the assembly using a sliding window of 200-bp. A filtering step was applied to include only the reads with high alignment scores. For Illumina reads, the cutoff was set at 200, which identified 120,804 reads corresponding to an average sequencing depth of 47-fold. For ONT reads, the cutoff was set at 1,000, which identified 10,562 reads corresponding to an average sequencing depth of 79-fold. (3) Potential mobile unit (PMU) regions (orange). (4) Two duplicated chromosomal segments (green; positions: 316–391 and 391–467 kb). [file Image_1.TIF]

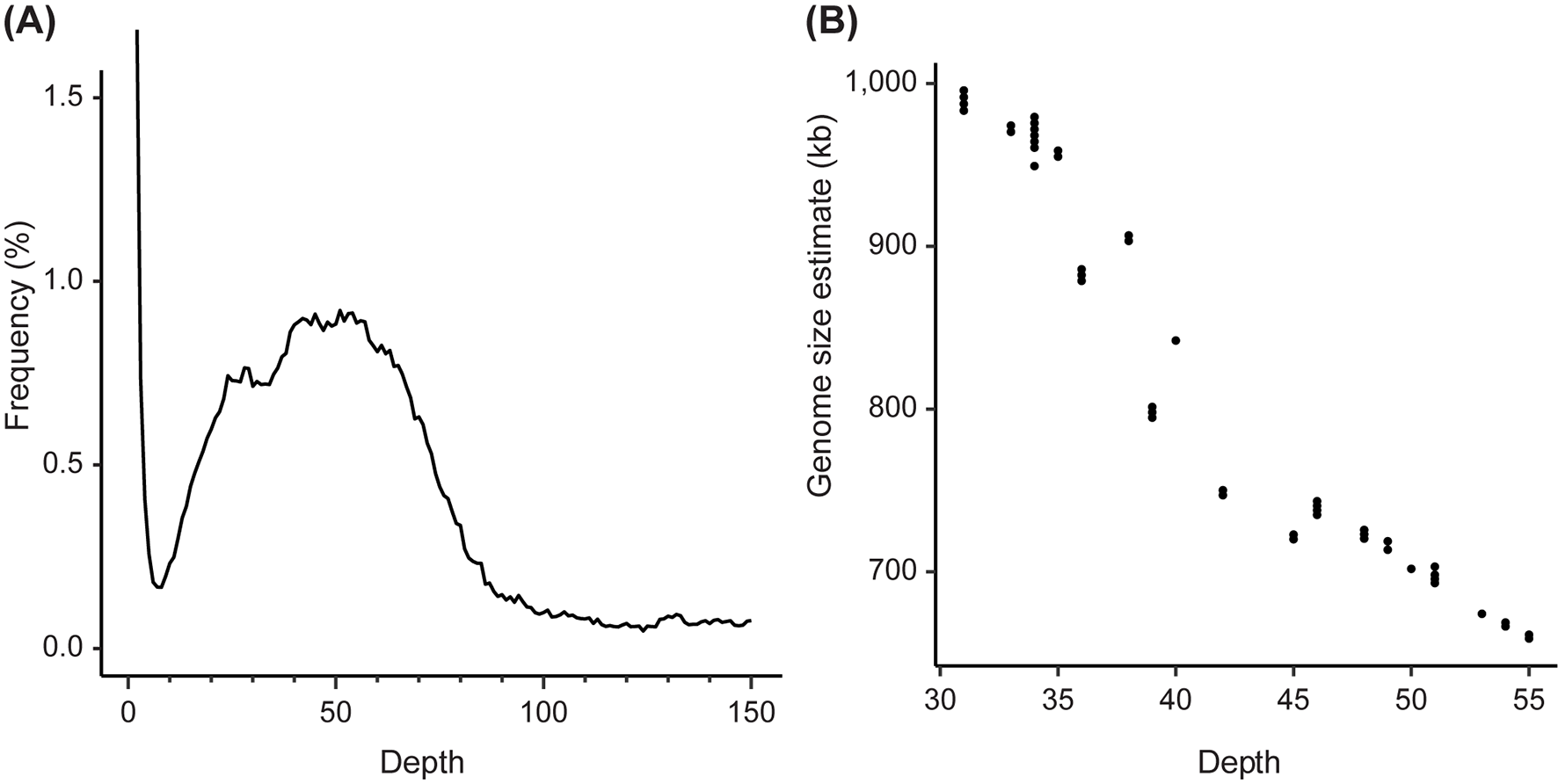

Supplement: Supplementary Figure 2 — Genome size estimation based on k-mer analysis. (A) A representative frequency distribution of k-mer depth (k = 21). The peak depth is 51. (B) Correspondence between genome size estimates and k-mer depths. [file Image_2.TIFF]

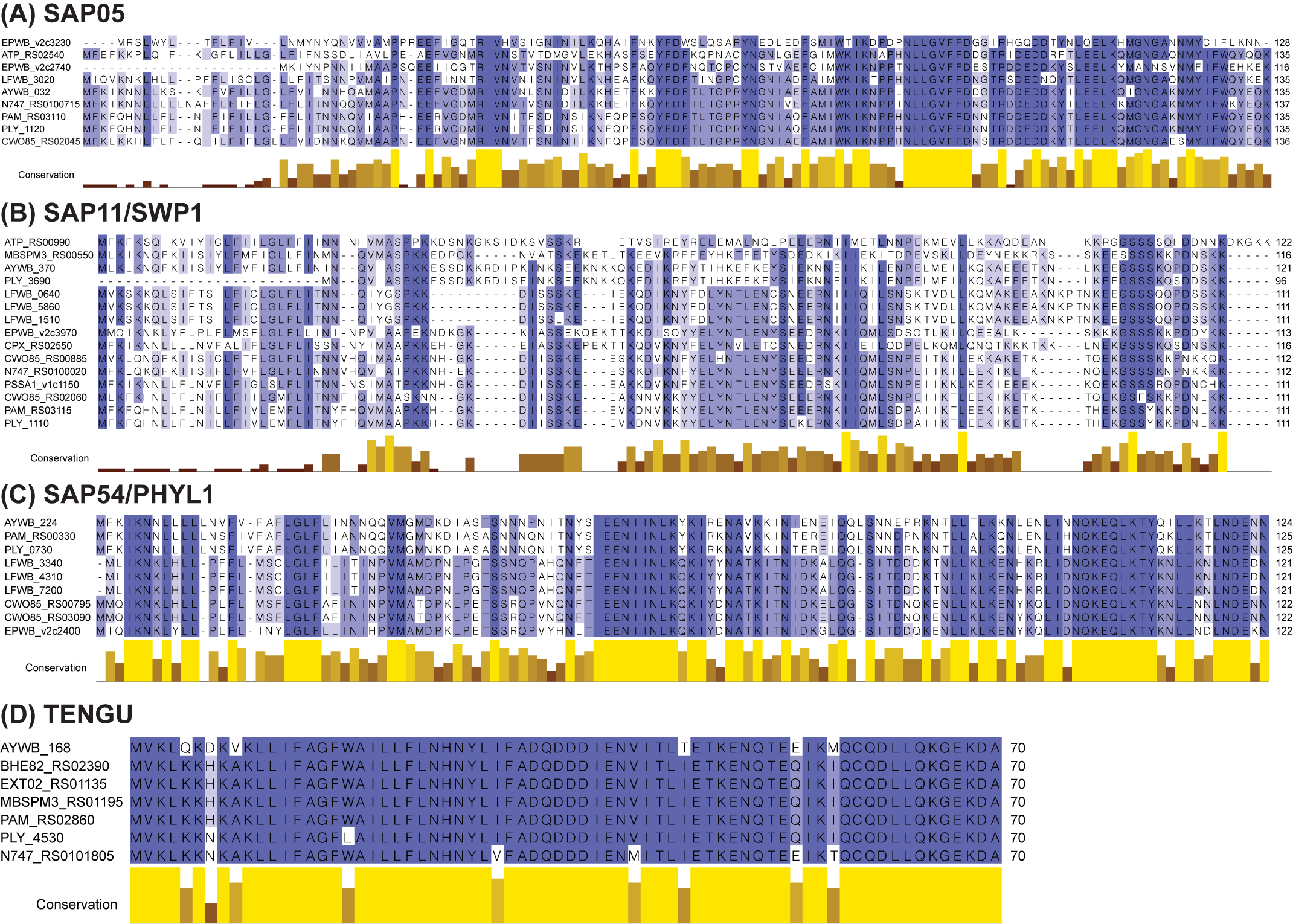

Supplement: Supplementary Figure 3 — Protein sequence alignments of four characterized phytoplasma effectors. Individual effectors are identified using locus tags. Shaded background colors indicate the levels of sequence conservation. (A) SAP05. (B) SAP11/SWP1. (C) SAP54/PHYL1. (D) TENGU. [file Image_3.TIFF]
